# Hierarchical Elemental Odor Coding for Fine Discrimination Between Enantiomer Odors or Cancer-Characteristic Odors

**DOI:** 10.3389/fnbeh.2022.849864

**Published:** 2022-04-22

**Authors:** Takaaki Sato, Mutsumi Matsukawa, Toshio Iijima, Yoichi Mizutani

**Affiliations:** ^1^Biomedical Research Institute, National Institute of Advanced Industrial Science and Technology, Osaka, Japan; ^2^Division of Anatomical Science, Department of Functional Morphology, Nihon University School of Medicine, Tokyo, Japan; ^3^Graduate School of Life Sciences, Tohoku University, Sendai, Japan; ^4^Department of Medical Engineering, Faculty of Health Science, Aino University, Osaka, Japan

**Keywords:** sniffer mouse behaviors, body odor disorder, biomarkers, fear and relaxation, odor discrimination, odor information coding, cancer-characteristic odors

## Abstract

Odors trigger various emotional responses such as fear of predator odors, aversion to disease or cancer odors, attraction to male/female odors, and appetitive behavior to delicious food odors. Odor information processing for fine odor discrimination, however, has remained difficult to address. The olfaction and color vision share common features that G protein-coupled receptors are the remote sensors. As different orange colors can be discriminated by distinct intensity ratios of elemental colors, such as yellow and red, odors are likely perceived as multiple elemental odors hierarchically that the intensities of elemental odors are in order of dominance. For example, in a mixture of rose and fox-unique predator odors, robust rose odor alleviates the fear of mice to predator odors. Moreover, although occult blood odor is stronger than bladder cancer-characteristic odor in urine samples, sniffer mice can discriminate bladder cancer odor in occult blood-positive urine samples. In forced-choice odor discrimination tasks for pairs of enantiomers or pairs of body odors vs. cancer-induced body odor disorders, sniffer mice discriminated against learned olfactory cues in a wide range of concentrations, where correct choice rates decreased in the Fechner's law, as perceptual ambiguity increased. In this mini-review, we summarize the current knowledge of how the olfactory system encodes and hierarchically decodes multiple elemental odors to control odor-driven behaviors.

## Introduction

The sense of smell functions as an extremely wide-ranging environmental sensor that informs us about approaches to odor sources of delicious or decayed foods, predators or others, healthy or diseased individuals, roses, other flowers, cedar trees, or camphor trees, etc. In addition, odors trigger various emotional responses such as fear to predator odors, aversion to disease or cancer odors, attraction to male/female odors, appetitive behavior to delicious food odors (for example for predator odor: Varnet-Maury et al., 1984; Fendt et al., 2003; Kobayakawa et al., 2007; Matsukawa et al., 2011; Murakami et al., 2012; Isosaka et al., 2015; Kondoh et al., 2016; Sato et al., 2017; for disease or cancer odors: Yamazaki et al., 2002; Matsumura et al., 2010; Sato et al., 2017; Gervasi et al., 2018; Sato et al., 2021). Odor information processing for fine odor discrimination, however, has remained difficult to address.

Sensory-guided behaviors, especially by the vision and/or olfaction in hazardous environments, are critical for survival and reproduction in animals including humans. Vision and olfaction share common features, which are as follows: (1) a set of G protein-coupled receptors (GPCRs) remotely discriminates and detects various color/odor stimuli, (2) sensory cells encode them into cellular signals with signal amplification via cyclic nucleotide second messenger systems and cyclic nucleotide-gated cation channels, and (3) elemental information in colors and odors would be extracted in the third neurons in the visual/olfactory pathway by addition and/or subtraction of signals between multiple receptors via inhibitory signals (for color vision: Sharpe et al., 1999; Burns and Lamb, 2004; Calkins, 2004; Coren et al., 2004; for olfaction: Ishikawa et al., 2007; Sato et al., 2008, 2014, 2016a, 2018; Sato, 2019). By analogy to color vision, we modeled odor information processing.

## Hierarchical Elemental Information Coding

### Hierarchical Elemental Color Coding

We can easily discriminate and find a red nandina fruit at the front of green leaves, whereas it is more difficult to find a small carrot on orange-colored leaves of Japanese maple. In the color vision, different orange colors can be discriminated by distinct intensity ratios of elemental colors, such as yellow and red. It is likely in a hierarchical elemental color-coding, where the dominancy of elemental colors is distinguished (Figure S1A). Four elemental colors (corresponding to the four primary colors) of blue (B), yellow (Y), green (G), and red (R) are essential to describe differences between distinct color hues in humans. The cone cells that detect color stimuli in different spectral sensitivities are the first cells in the visual pathway (Calkins, 2004). There are only three types of cone cells, the short (S)-, middle (M)-, and long (L)-wavelength sensitive in the retina (Figures S1B,C) (Sharpe et al., 1999; Calkins, 2004; Coren et al., 2004). The retinal ganglion cells, which are the third cells in the visual pathway, represent the four elemental colors in the two channels of Y/B and R/G (Calkins, 2004; Coren et al., 2004). A schematic diagram indicates how four elemental colors of Y/B and R/G are extracted in the ganglion cells by addition and subtraction of signals between three L, M, and S signals via inhibitory signals (Figure S1C) (Sharpe et al., 1999; Calkins, 2004; Coren et al., 2004). B, G, and R are S-, M-, and L-unique elemental colors, respectively, whereas Y is the M- and L-common elemental color being represented by the addition of signals from L and M.

See the remaining parts in Supplementary Material.

### Hierarchical Elemental Odor Coding

**Figure 1:**
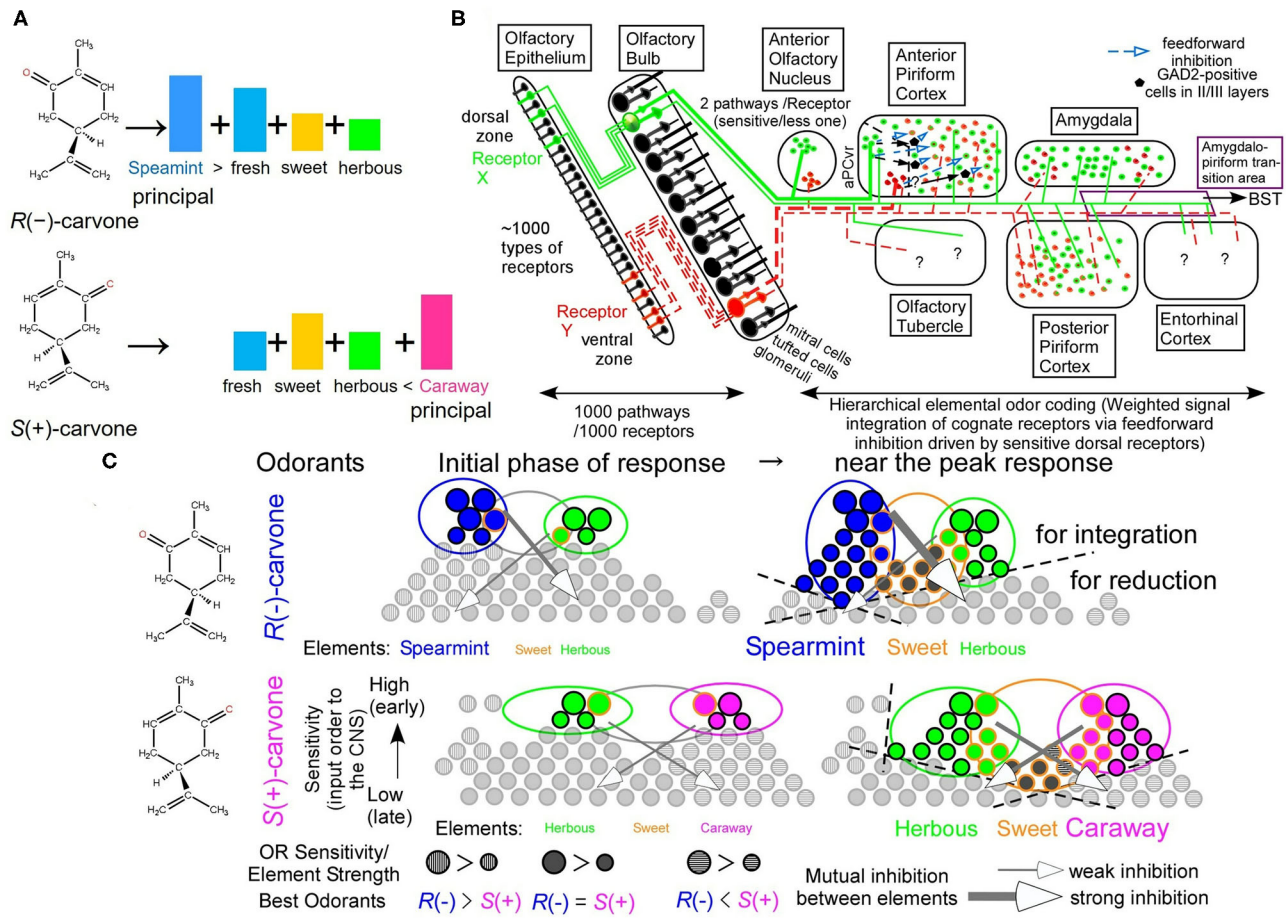
Hierarchical elemental odor coding in olfaction. **(A)** Schematic diagram of similar but different odors of carvone enantiomers in a hierarchical elemental odor coding. Predicted relative intensities of elemental odors differ between the enantiomers. **(B)** Olfactory pathway from the olfactory epithelium to the olfactory cortex (Modified from Sato et al., 2008). Pyramidal cells in the anterior piriform cortex, where elemental odors are likely represented by addition and subtraction between signals from multiple receptors via the feedforward inhibitory signals, are the third neurons from sensory neurons (Sato et al., 2007, 2014). Predator odors activate the amygdala, the bed nucleus of the stria terminalis (BST), and the anterior pituitary resulting in an increase of adrenocorticotrophic hormone (ACTH) to reduce fear stress (see Matsukawa's review in this collection). **(C)** Schematic diagram of receptor signal integrations for principal elemental odors among cognate receptors by inhibitory signals (Sato et al., 2007, 2014, 2016c). These are the cases of carvone enantiomers at the initial phase of odor response and around the peak of receptor responses (See text for the details).

Enantiomeric pairs of mirror-image molecular structures are difficult to resolve by instrumental analyses. The human olfactory system, however, discriminates *R*(–)-carvone from its (+)-form rapidly within seconds. As different relative strengths of Y and R elemental colors for different orange colors, odors of the carvone enantiomers differ in relative intensities of overlapped elemental odors of sweet, fresh, and herbous (Figure 1A). *R*(–)-carvone is fresher than *S*(+)-carvone, whereas *S*(+)-carvone is sweeter than *R*(–)-carvone. In addition, the most prominent elemental odors of *R*(–)- and *S*(+)-carvones are uniquely spearmint-like and caraway-like odors, respectively. In a hierarchical elemental odor coding, differences in the principal elemental odors and/or relative elemental odor profiles allow easy discrimination between similar but spearmint-like and caraway-like odors. Similar to color vision, some evidence indicates that elemental odors are extracted by addition and subtraction of signals between cognate receptors and non-cognate receptors in the 3rd neurons via inhibitory signals (Ishikawa et al., 2007; Sato et al., 2007, 2016a,b,c, 2018; Matsukawa et al., 2011; Sato, 2019).

A schematic diagram of the main olfactory pathway from the first neurons to the third neurons is illustrated in Figure 1B. In mice, there are ca. 1,130 types of olfactory receptors (ORs), which are alternatively expressed in olfactory sensory neurons (OSNs) in either of the dorsal or ventral zone in a manner of one neuron–one receptor (Malnic et al., 1999; Serizawa et al., 2004). Axonal projection of OSNs expressing a given type of ORs converges to one or two glomeruli in the olfactory bulb (OB) in a manner of one glomerulus–one receptor (Mombaerts et al., 1996), likely for an improved signal-to-noise ratio by integration of signals from the identical ORs. At the glomerulus, the olfactory pathway for signals from given ORs separates into two routes, a sensitive signal pathway via tufted cells and a less-sensitive and lateral inhibition-mediated sharper tuning signal pathway via mitral cells (Igarashi et al., 2012). Neighboring configuration of glomeruli for signals from ORs for structurally-resemble odorants (Takahashi et al., 2004) would facilitate sharpening their overlapped tuning specificities via lateral inhibition from periglomerular cells and/or granule cells in the OB. This is a part of the decorrelation of strongly overlapped OR signals in postsynaptic circuitry in the OB (Friedrich et al., 2009; Niessing and Friedrich, 2010; Wiechert et al., 2010; Gilra and Bhalla, 2015).

There are seven olfactory cortical areas, which directly receive output signals from the OB tufted/mitral cells, the anterior olfactory nucleus (AON), the anterior piriform cortex (aPC), the posterior piriform cortex (pPC), the amygdala (AMY), the amygdalo-piriform transition area (Amy-PirTA), the entorhinal cortex (EC), and the olfactory tubercle (OT) (Figure 1B). Among them, only the AON and the ventro-rostral region of the aPC (aPCvr) receive the output signals from the OB tufted cells via the sensitive pathway (Haberly and Price, 1977; Matsutani et al., 1989; Ekstrand et al., 2001; Igarashi et al., 2012). In addition, only in the aPC, odor responses immediately develop a much greater inhibitory component (surface-positive local field potentials) of the feedforward inhibitory signals from the aPCvr compared to an initial small excitatory component (surface-negative local field potentials) (Ishikawa et al., 2007; Sato et al., 2008). Like the extraction process of the Y elemental color by the addition of L and M signals and subtraction of S signals, the feedforward inhibitory signals in the aPC could drive stimulus-characteristic elemental odor-enhancing hierarchical odor coding by input synchrony between identical OR signals and between cognate OR signals from mitral cells after mutual inhibition (Sato et al., 2008, 2014, 2015, 2016a, 2018).

We have found direct evidence for the feedforward inhibition-driven hierarchical enhancement of a given elemental odor in an odor mixture. It is well-known that a fox-unique compound, 2,5-dihydro-2,4,5-trimethylthiazoline (TMT), induces fear stress responses in mice as an olfactory cue of the predator, increasing in plasma adrenocorticotrophic hormone (ACTH). Signals from ORs for TMT are sent to the bed nucleus of stria terminalis (BST) via the Amy-PirTA for releasing plasma ACTH (Kondoh et al., 2016). Surprisingly, a rose odor alleviates the fear stress of mice to the life-threatening TMT odor via a reduced feedforward inhibition (Matsukawa et al., 2011, see Matsukawa's review in this collection). This result can be interpreted as rose odor-induced feedforward inhibition-driven rose odor enhancement by input synchrony but not for TMT odor enhancement. This also suggests that a present of a bouquet of old roses is a good plan to relieve a female's stress on the first date.

In addition, a novel method using wavelet time-frequency power profiles revealed a change in odor information redundancy in the aPC pyramidal cells by the addition of multiple receptor signals, that is, a change from “an odor experience-dependent correlation in input signals to the aPC pyramidal cells” to “an odor identity-dependent correlation in output signals from the aPC pyramidal cells” (Sato et al., 2016a). Thus, the aPC pyramidal cells correspond to the retinal ganglion cells for extracting elemental colors or odors in the third neurons in the sensory pathway.

Regarding temporal order-dependency of information processing by input signals of activated ORs to carvone enantiomers, in the initial phase of response, the most sensitive ORs for a given carvone enantiomer are first activated and integrated with the aPC pyramidal cells via feedforward inhibition, resulting in clearly different elemental odor profiles with a partial overlap between the two carvone enantiomers (Figure 1C) (Sato et al., 2014, 2015, 2016c, 2018). Around the peak of the response, much more ORs are activated and contribute to enantiomer-characteristic elemental odors more than enantiomer-common elemental odors by input synchrony-driven OR signal integrations and mutual inhibitions, resulting in auxiliary weaker and common elemental odors adding to prominent odorant-characteristic elemental odors (the right part of Figure 1C). This stimulus-driven characteristic elemental odor enhancing system likely enables us to easily discriminate between various similar odors (Hamana et al., 2003).

See the remaining parts in Supplementary Material.

## Genetic Ablation of all Dorsal Olfactory Receptors Impairs Enantiomer Odor Discrimination and Sensitivities to Some Odorants

Next, we examined the effects of OR deletion on odor discrimination. Wild-type (WT) mice displayed an exquisite “supersensitive detection” (black star) and similar “supersensitive discrimination” (black bar) to enantiomeric odor pairs of wine lactones and carvones in an odor plume-guided Y-maze behavioral assays (Figure S2A) (Sato et al., 2015). Surprisingly, by genetic ablation of all dorsal ORs, ΔD mice retained the supersensitivity to *R*(–)-carvone and selectively reduced sensitivity to *S*(+)-carvone with a 10^4^-fold higher detection threshold (red star). More surprisingly, ΔD mice displayed an extremely reduced discrimination sensitivity for *R*(–)- vs. *S*(+)-carvone odors with a 10^10^-fold higher discrimination threshold (red bar) compared to those of WT mice (Figure S2A) (Sato et al., 2015). The similarly high detection sensitivity of ΔD mice for *R*(–)-carvone indicates the existence of highly sensitive ventral ORs, which are yet identified as well as the most sensitive dorsal ORs to *S*(+)-carvone. Nevertheless, this impaired odor discrimination for *R*(–)- vs. *S*(+)-carvone could not be explained by the combinatorial receptor coding scheme (Malnic et al., 1999), because receptor codes for *R*(–)- and *S*(+)-carvones definitely differ each other even after deletion of all dorsal ORs (marked by the cross) (ventral OR codes without the cross mark in Figures S2C,D). This is a discrimination paradox in the combinatorial receptor coding scheme (Sato et al., 2015). Instead, the hierarchical elemental odor coding scheme enables to explain these unusual changes in thresholds for enantiomeric odor detection and discrimination by genetic ablation of all dorsal ORs. As described in Supplementary Material, the dorsal helix-8-2^nd^-Glu car-5^*^ OR would be a key OR for enhancing *R*(–)-carvone-unique elemental odors via initially activated feedforward inhibitory signals. If this is the case, the deletion of the key OR, the dorsal helix-8-2^nd^-Glu car-5^*^, likely leads to impair to represent a prominent or robust auxiliary *R*(–)-carvone-unique spearmint-like odor in the aPC pyramidal cells and results in prominent *R*(–)-/*S*(+)-carvone-common sweet and herbous odors via signals from car-*266* OR (enclosed in the orange rectangle) and its cognate ventral ORs. Ventral ORs would be less sensitive to *S*(+)-carvone than the most sensitive dorsal ORs. Thus, the remaining less sensitive ventral ORs are unlikely to represent robust signals of *S*(+)-carvone-unique caraway-like odor via feedforward inhibitory signals. Moreover, ΔD mice also retain a similar sensitivity to TMT as WT mice but cannot recognize the TMT odor as a predator-characteristic odor, resulting in no fear response (Kobayakawa et al., 2007). Our model provides a more nuanced resolution of the enantiomer odor discrimination paradox.

See additional supporting evidence in Supplementary Material.

## Bladder- and Prostate-Cancer Odor Detection and Discrimination

**Figure 2:**
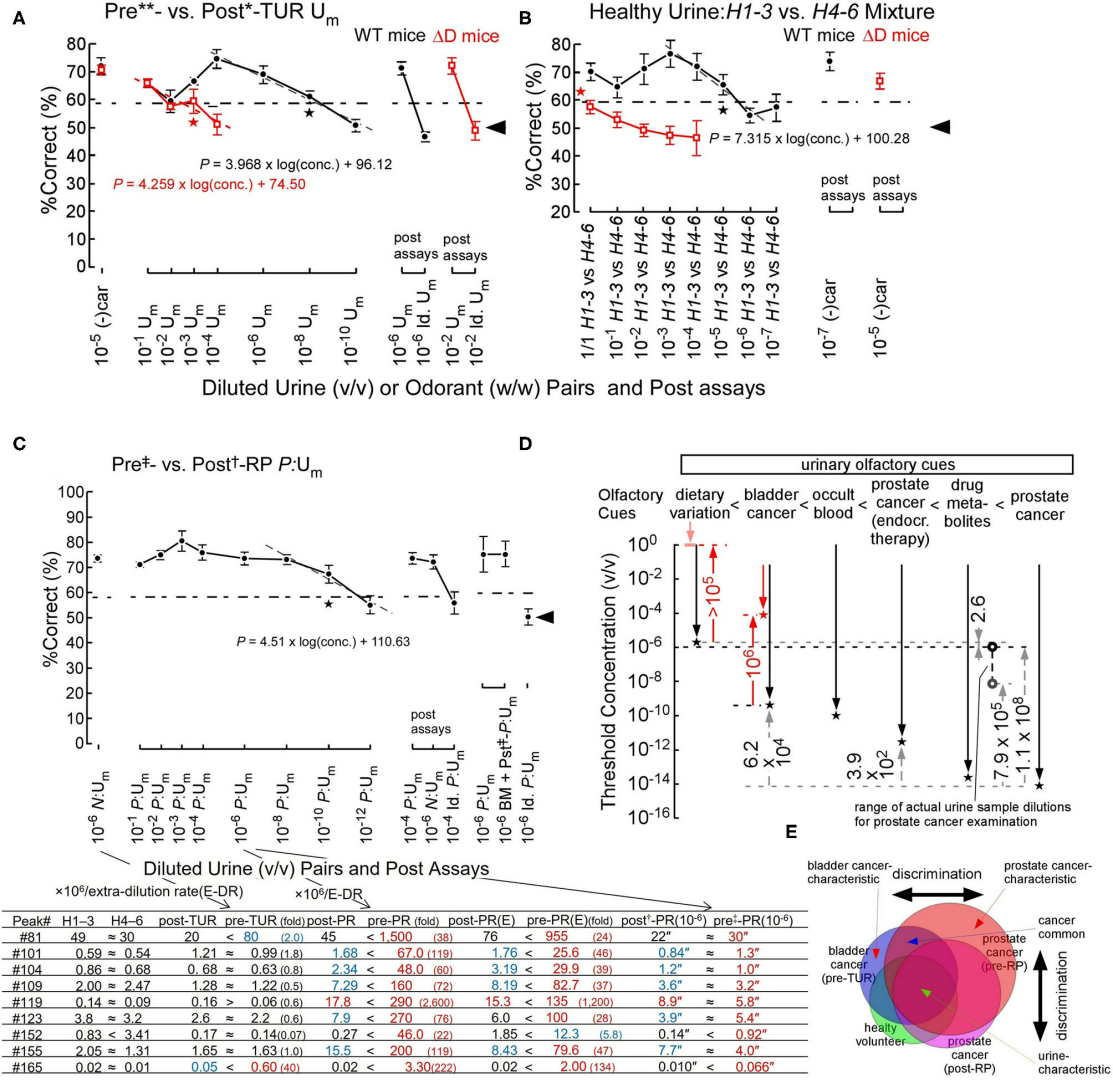
Odor discrimination thresholds of wild-type (WT) and ΔD mice for bladder and prostate cancers and healthy volunteer dietary variation (Sato et al., 2017, 2021). **(A)** Odor discrimination of WT (black closed circles) and ΔD mice (red open squares) between equi-occult blood pre**- vs. post*-transurethral resection (post*-TUR) urine mixture (U_m_) of five patients with bladder cancer (Sato et al., 2017). ΔD mice showed a marked elevation of discrimination threshold for bladder cancer odor (pre-TUR odor) vs. post-TUR odor. Post-assays, 10^−6^ pre**- vs. post*-TUR U_m_ and identical U_m_ pair: 10^−6^ pre**- vs. pre**-TUR U_m_. The percent correct (%Correct) for a training odor pair−10^−5^ R-(–) carvone [(–)car] (w/w) vs. solvent [di(propylene)glycol] just before the urine discrimination assay is shown on the left side. Linear regression models are shown around the thresholds (gray or red dashed lines). Two alternative forced choice assays with target vs. non-target odors were performed in a Y-maze. Tasks performed at thresholds are marked by the star. Chain lines indicate the %Correct significantly above chance performance (*P* = 0.05). Black arrowheads indicate chance levels (50%). **(B)** Urine odor discrimination between a pair of healthy (*H*) urine mixtures—six volunteers' 1^st^-3^rd^ sample (*H*1–3) vs. 4^th^-6^th^ sample (*H*4–6) U_m_. ΔD mice could not discriminate the healthy volunteer urine pair. Post assays: 10^−7^ and 10^−5^ (–)car vs. solvent for WT and ΔD mice, respectively. **(C)** Odor discrimination of sniffer mice between equal-occult blood pre^‡^- vs. post^†^-radical prostatectomy (post^†^-RP) U_m_ of five patients with prostate cancer (Sato et al., 2021). Post-assays, 10^−4^ *P*:U_m_ pair: 10^−4^ pre^‡^- vs. post^†^-RP *P*:U_m_, 10^−6^ *N*:U_m_ pair: pre**- vs. post*-TUR of bladder cancer *N*:U_m_ and identical *P*:U_m_ pair: 10^−4^ pre^‡^- vs. pre^‡^-RP *P*:U_m_. The percent correct (%Correct) for a training odor pair−10^−6^ *N*:U_m_ pair just before the prostate cancer urine discrimination assay is shown on the left side. On the right side, the identical %Correct between pairs of pre^‡^- vs. post^†^-RP U_m_ and post^‡^-RP + biomarker (BM) U_m_ (prostate cancer odor mimic) vs. post^†^-RP U_m_, and by chance choice for identical pre^‡^-RP U_m_ pair are shown. Extra-dilution rates for equal-occult blood U_m_s are 1/13** v/v, 1/6* v/v, 1/50^‡^ v/v, and 1/2^†^ v/v. The lower panel shows concentrations (ppb) (-fold of healthy control) of respective peak compounds in the respective original samples, and concentration″ (ppq) in the (10^6^ × extra rate)-fold diluted urine samples. **(E)** After neoadjuvant endocrine therapy. “≈” means <2 ratios of paired sample concentrations. Biomarker compounds for peaks are #81, phenol; #101, dimethyl succinate; #104, acetophenone; #109, 2-phyenyl-2-propanol; #119, 3,5,5-trimethyl-2-cyclohexenone; #123, dimethyl glutarate; #152, piperitone; #155, 2-hydroxy-2-methylpropiophenone; #165, 2,6-di(propan-2-yl)phenol. **(D)** Odor discrimination thresholds of sniffer mice for urinary olfactory cues (Sato et al., 2017, 2021). Odor discrimination ranges (downward arrows) and thresholds (stars) of WT (black plots) and ΔD mice (red plots) for urinary olfactory cues are shown. Observed threshold differences indicate that urinary olfactory cues increase in the following order: dietary variation < bladder cancer < occult blood < prostate cancer (after neoadjuvant endocrine therapy) < antibiotic drug metabolites < prostate cancer. ΔD mice exhibited reduced odor discrimination sensitivities, compared to WT mice; degrees of sensitivity reductions due to ablation of dorsal olfactory receptors are indicated by the red upward arrows. Range of actual concentrations of urine mixture samples for prostate cancer examination in 10^6^-fold diluted equal-occult blood conditions ranged from 1.0 × 10^−6^ v/v (black open circle) to 7.1 × 10^−9^ v/v (gray open circle). **(E)** Schematic diagram for discrimination between similar but distinct cancer odors (Sato et al., 2021). The sniffer mice would discriminate disease-biomarker odors based on the relative ratio of biomarkers for cancers (cancer-characteristic + cancer-common compounds) vs. urine-characteristic compounds. Overlapping regions contain compounds common to different cancers or cancers and healthy variations, leading to odor similarity, whereas non-overlapping regions contain status-characteristic compounds, leading to an odor-cue mismatch.

Next, we asked how sensitively sniffer mice can discriminate urinary olfactory cues such as genetically determined body odors, diet-modified body odors, and cancer-induced body odor disorders. In forced-choice odor discrimination, behavioral assays in an odor plume-guided Y-maze, sniffer mice showed odor discrimination thresholds of 5.7 × 10^−10^ v/v and 2.6 × 10^−6^ v/v for bladder cancer odor and dietary variations of body odors in healthy volunteers, respectively (on the gray dashed line of a linear regression model in Figures 2A,B) (Sato et al., 2017). The %Correct of odor choice for a learned olfactory cue decreased semi-logarithmically in Fechner's law around the threshold, as the concentration of urine sample decreased (Figure 2). This decrease in %Correct of odor choice indicates a semi-logarithmic increase in perceptual ambiguity of sniffer mice in odor discrimination.

Interestingly, the genetic ablation of all dorsal ORs elevated odor discrimination thresholds (2.9 × 10^−4^ v/v) for bladder cancer odor by 5 × 10^5^-fold (on the red dashed line of a linear regression model in Figure 2A) (Sato et al., 2017). In addition, ΔD mice also showed a >10^5^-fold elevation in odor discrimination threshold for healthy volunteers' dietary variations of body odors, resulting in the inability of ΔD mice to discriminate the urinary odor pair of healthy volunteers (Figure 2B) (Sato et al., 2017). These great reductions in body odor discrimination sensitivity by the dorsal OR ablation again support a hierarchical enhancement of body odor-characteristic elemental odor via feedforward inhibition activated by dorsal ORs. Notably, odor detection threshold of ΔD mice was elevated by 10^4^-fold for *n*-hexanoic acid, to which dorsal class-I ORs are sensitive, whereas that of ΔD for *n*-decanoic acid, to which ventral ORs are sensitive, was similar to that of WT mice (our unpublished data).

An olfactory cue of prostate cancer odor resulted in an extremely low odor discrimination threshold of 9.2 × 10^−15^ v/v (on the gray dashed line in Figure 2C) (Sato et al., 2021). A linear regression model of %Correct vs. log(conc.) around the threshold again indicates a semi-logarithmically increased perceptual ambiguity of sniffer mice in odor discrimination (Figure 2C). We found eight biomarkers with different profiles for prostate and bladder cancers and validated the biomarker profile as the olfactory cue of prostate cancer odor even in a urine sample from patients after radical prostatectomy (BM + pst^‡^-*P*:U_m_, Figure 2C) (Sato et al., 2021). In the post assays, an identical odor pair (Id. *P*:U_m_) resulted in odor choice at the chance level and the learned odor pair made the sniffer mice possible to discriminate them again at a high correct rate, although the urinary biomarkers were at similar concentrations of ppq between pre^‡^- and post^†^-RP *P*:U_m_. These post assays confirmed that the sniffer mice basically choose one of the Y-maze branches by olfactory cues but not by visual cues. In addition, the prostate cancer-characteristic odor would be perceived as relatively stronger elemental odors compared to urine-common elemental odors (Figure 2E) (Sato et al., 2021). In fact, prostate cancer-induced 22- to 2,600-fold increases in nine biomarker concentrations, whereas bladder cancer induced 2- to 40-fold increases only in two biomarkers (the lower panel of Figure 2C) (Sato et al., 2021). Prostate or bladder cancer-characteristic odors would be represented and automatically enhanced in a hierarchical elemental odor coding scheme by feedforward inhibition activated by signals from helix-8-2^nd^-Glu ORs most sensitive to some of the nine biomarkers. Notably, our results suggested that a neoadjuvant endocrine therapy reduced the prostate cancer odor and the biomarker concentrations by 2,500-fold (for threshold concentrations) and 1.6–3.8-fold, respectively, suggesting a reduced tumor volume.

Finally, our data indicate that intensities of urinary olfactory cues increase in the order of dietary variation < bladder cancer < occult blood < prostate cancer after neoadjuvant endocrine therapy < antibiotic drug metabolites < prostate cancer (Figure 2D) (Sato et al., 2021). Although a genetically determined body odor (like body odor fingerprints) is weaker than dietary variation in mice, sniffer mice can discriminate such a weak olfactory cue in a urine sample (Schaefer et al., 2002; Kwak et al., 2008).

## Shared Features Between Humans and Mice

The helix-8 2^nd^ residues of ORs are 91% (248/273) identical between humans and mice in class-I [93% (39/42), Glu and Gln] and class-II [90% (204/226), Glu, Gln, and Asp] ORs, and TAARs [100% (5/5), Trp] (Sato et al., 2018). See additional information and the shared elemental odors of vanilla, creamy and cinnamon between murine OR codes and human perception (Furudono et al., 2009) in Supplementary Material.

## Future Studies

The olfactory system is comprised of the most complicated neural network. There are more than hundreds of ORs with one of helix-8-2^nd^-Glu, Gln, Asp, Trp, and minor Lys/His (for off-response?), which would differ in specific interaction with G_α*olf*_ or G_α*i*_ leading to different rapidity and robustness of cellular responses in OSNs. In addition, quite similar or different odorants-activated OR signals are processed separately and/or interactively in seven distinct olfactory cortical areas. In this odor information processing, one of the key processes is a constantly rapid and specific interaction between ORs and G_α*olf*_, because variable binding kinetics of OR–G_α*olf*_ interaction could cause changes in an order of signal inputs from ORs to the brain between higher and lower affinities to a given odorant resulting in unlikely sniffing-by-sniffing variation in odor representations. Type-specific stable interaction sites in the C-terminal region of G protein have been proposed (Flock et al., 2017), but counterpart residues of GPCRs have been not identified (Sato, 2019). Compared to entirely low sequence homology of GPCRs, the highly conserved helix-8 2^nd^ residue in groups for identical best ligands and specific G protein types would confer distinct functional roles of helix-8-2^nd^-residue classified GPCRs in their signaling pathways. Based on these principles, we will understand the details of the complicated odor information processing for behavioral controls in the olfactory system with decorrelation of strongly overlapped OR signals in postsynaptic circuitry in the OB and the sensitive and less-sensitive OR signal routes from the OB to the aPC (Matsutani et al., 1989; Igarashi et al., 2012). See an additional part in Supplementary Material.

## Author Contributions

TS planned and conducted most of the project with coauthors. MM planned and conducted experiments for the effects of different odors on fear stress responses of mice to TMT odor. TI planned and conducted experiments for the identification of sources of feedforward inhibition in the aPC in isolate guinea-pig whole brain. YM planned and conducted a collection of urine samples from patients and healthy volunteers before and after tumor resection. TS and MM wrote the manuscript. All authors discussed and analyzed the results. All authors contributed to the article and approved the submitted version.

## Funding

This work was funded by Grant-in-Aids for Scientific Research (B) (#22300066, #18300066, and #15H02730 to TS), Grantin-Aids for Scientific Research (C) (#20500195, #23500264, #15K00381, #18K11507, and #21K12092 to MM), Nihon University Research Grant (#kyo09-010 to MM), the Human Frontier Science Program grant n-RG19/96 to TI, grants from Aino University to YM, and research budget from Sumitomo Chemical Co., Ltd for SPME-GC-MSmeasurements. The authors declare that this study received funding from Japan Tobacco INC.

## Conflict of Interest

The authors declare that this study received research budget from Sumitomo Chemical Co., Ltd, which was used for SPME-GC-MS measurements. This study received funding from Japan Tobacco INC. The funder had the following involvement in the study: Shared features of odor coding between humans and mice.

## Publisher's Note

All claims expressed in this article are solely those of the authors and do not necessarily represent those of their affiliated organizations, or those of the publisher, the editors and the reviewers. Any product that may be evaluated in this article, or claim that may be made by its manufacturer, is not guaranteed or endorsed by the publisher.

## References

[B1] Burns M. E. Lamb T. D. (2004). Visual transduction by rod and cone photoreceptors, in The Visual Neurosciences, eds Chalupa L. M. Werner J. S. (Cambridge, MA: MIT Press), 215–233.

[B2] Calkins D. J. (2004). Linking retinal circuits to color opponency, in The Visual Neurosciences, eds Chalupa L. M. Werner J. S. (Cambridge, MA: MIT Press), 989–1002.

[B3] Coren S. Ward L. M. Enns J. T. (2004). Chapter 4: Brightness and color, in Sensation and Perception, 6th Edn (Hoboken, NJ: John Wiley and Sons, Inc.), 80–115.

[B4] Ekstrand J. J. Domroese M. E. Johnson D. M. Feig S. L. Knodel S. M. Behan M.. (2001). A new subdivision of anterior piriform cortex and associated deep nucleus with novel features of interest for olfaction and epilepsy. J. Comp. Neurol. 434, 289–307. 10.1002/cne.1178 11331530

[B5] Fendt M. Endres T. Apfelbach R. (2003). Temporary inactivation of the bed nucleus of stria terminalis but not of the amygdala blocks freezing induced by trimethylthaizoline, a component of fox feces. J. Neurosci. 23, 23–28. 10.1523/JNEUROSCI.23-01-00023.2003 12514197 PMC6742150

[B6] Flock T. Hauser A. S. Lund N. Gloriam D. E. Balaji S. Babu M. M. (2017). Selectivity determinants of GPCR–G-protein binding. Nature 545, 317–322. 10.1038/nature22070 28489817 PMC5846738

[B7] Friedrich R. W. Yaksi E. Judkewitz B. Wiechert M. T. (2009). Processing of odor representations by neuronal circuits in the olfactory bulb. Ann. N. Y. Acad. Sci. 1170, 293–297. 10.1111/j.1749-6632.2009.04010.x 19686150

[B8] Furudono Y. Sone Y. Takizawa K. Hirono J. Sato T. (2009). Relationship between peripheral receptor code and perceived odor quality. Chem. Senses 31, 151–159. 10.1093/chemse/bjn071 19073951

[B9] Gervasi S. S. Opiekun M. Martin T. Beauchamp G. K. Kimball B. A. (2018). Sharing an environment with sick conspecifics alters odors of healthy animals. Sci. Rep. 8, 14255. 10.1038/s41598-018-32619-4 30250285 PMC6155122

[B10] Gilra A. Bhalla U. S. (2015). Bulbar microcircuit model predicts connectivity and roles of interneurons of odor coding. PLoS ONE 10, e0098045. 10.1371/journal.pone.0098045 25942312 PMC4420273

[B11] Haberly L. B. Price J. L. (1977). The axonal projection patterns of the mitral and tufted cells of the olfactory bulb in the rat. Brain Res. 129, 152–157. 10.1016/0006-8993(77)9097807 68803

[B12] Hamana H. Hirono J. Kizumi M. Sato T. (2003). Sensitivity-dependent hierarchical receptor codes for odors. Chem. Senses 28, 87–104. 10.1093/chemse/28.2.87 12588732

[B13] Igarashi K. M. Ieki N. An M. Yamaguchi Y. Nagayama S. Kobayakawa K.. (2012). Parallel mitral and tufted cell pathways route distinct odor information to different targets in the olfactory cortex. J. Neurosci. 32, 7870–7885. 10.1523/JNEUROSCI.0154-12.2012 22674272 PMC3636718

[B14] Ishikawa T. Sato T. Shimizu A. Tsutsui K. de Curtis M. Iijima T. (2007). Odor-driven activity in the olfactory cortex of an *in vitro* isolated guinea pig whole brain with olfactory epithelium. J. Neurophysiol. 97, 670–679. 10.1152/jn.01366.2005 16870834

[B15] Isosaka T. Matsuo T. Yamaguchi T. Funabiki K. Nakanishi S. Kobayakawa R.. (2015). Htr2a-expressing cells in the central amygdala control the hierarchy between innate and learned fear. Cell 163, 1153–1164. 10.1016/j.cell.2015.10.047 26590419

[B16] Kobayakawa K. Kobayakawa R. Matsumoto H. Oka Y. Imai T. Ikawa M.. (2007). Innate versus learned odour processing in the mouse olfactory bulb. Nature 450, 503–508. 10.1038/nature06281 17989651

[B17] Kondoh K. Lu Z. Ye X. Olson D. P. Lowell B. B. Buck L. B. (2016). A specific area of olfactory cortex involved in stress hormone responses to predator odors. Nature 532, 103–106. 10.1038/nature17156 27001694 PMC5094457

[B18] Kwak J. Willse A. Matsumura K. Curran Opiekun M. Yi W. Preti G.. (2008). Genetically-based olfactory signatures persist despite dietary variation. PLoS ONE 3, e3591. 10.1371/journal.pone.0003591 18974891 PMC2571990

[B19] Malnic B. Hirono J. Sato T. Buck L. (1999). Combinatorial receptor codes for odors. Cell 96, 713–723. 10.1016/s0092-8674(00)80581-4 10089886

[B20] Matsukawa M. Imada M. Murakami T. Aizawa S. Sato T. (2011). Rose odor can innately counteract predator odor. Brain Res. 1381, 117–123. 10.1016/j.brainres.2011.01.053 21266167

[B21] Matsumura K. Opiekun M. Oka H. Vachani A. Albelda S. M. Yamazaki K.. (2010). Urinary volatile compounds as biomarkers for lung cancer: a proof of principle study using odor signatures in mouse models of lung cancer. PLoS ONE 5, e8819. 10.1371/journal.pone.0008819 20111698 PMC2811722

[B22] Matsutani S. Senba E. Tohyama M. (1989). Terminal field of cholecystokinin-8-like immunoreactive projection neurons of the rat main olfactory bulb. J. Comp. Neurol. 285, 73–82. 10.1002/cne.902850107 2754048

[B23] Mombaerts P. Wang F. Dulac C. Chao S. K. Nemes A. Mendelsohn M.. (1996). Visualizing an olfactory sensory map. Cell 87, 675–686. 10.1016/S0092-8674(00)81387-2 8929536

[B24] Murakami T. Matsukawa M. Katsuyama N. Imada M. Aizawa S. Sato T. (2012). Stress-related activities induced by predator odor may become indistinguishable by hinokitiol odor. Neuroreport 23, 1071–1076. 10.1097/WNR.0b013e32835b373b 23128452

[B25] Niessing J. Friedrich R. W. (2010). Olfactory pattern classification by discrete neuronal network states. Nature 465, 47–52. 10.1038/nature08961 20393466

[B26] Sato T. (2019). Conserved 2nd residue of helix 8 of GPCR may confer the subclass-characteristic and distinct roles through a rapid initial interaction with specific G proteins. Int. J. Mol. Sci. 20, 1752. 10.3390/ijms20071752 30970644 PMC6480185

[B27] Sato T. Hirono J. Hamana H. Ishikawa T. Shimizu A. Takashima I.. (2008). Architecture of odor information processing in the olfactory system. Anat. Sci. Int. 83, 195–206. 10.1111/j.1447-073X.2007.00215.x 19159347

[B28] Sato T. Ishikawa T. Shimizu A. Hirono J. Hamana H. Iijima T. (2007). Molecular basis of odor discrimination in olfactory system. Seitai-no-Kagaku 58, 264–268. 10.11477/mf.2425100044

[B29] Sato T. Kajiwara R. Takashima I. Iijima T. (2016a). A novel method for quantifying similarities between oscillatory neural responses in wavelet time-frequency power profiles. Brain Res. 1636, 107–117. 10.1016/j.brainres.2016.01.054 26855257

[B30] Sato T. Katsuoka Y. Yoneda K. Nonomura M. Uchimoto S. Kobayakawa R.. (2017). Sniffer mice discriminate urine odors of patients with bladder cancer: a proof-of-principle study for non-invasive diagnosis of cancer-induced odors. Sci. Rep. 7, 14628. 10.1038/s41598-017-15355-z 29116175 PMC5676727

[B31] Sato T. Kawasaki T. Mine S. Matsumura H. (2016b). Functional role of the C-terminal amphipathic helix 8 of olfactory receptors and other G protein-coupled receptors. Int. J. Mol. Sci. 17, E1930. 10.3390/ijms17111930 27869740 PMC5133925

[B32] Sato T. Kobayakawa R. Kobayakawa K. Emura M. Itohara S. Kawasaki T.. (2016c). Supersensitive odor discrimination is controlled in part by initial transient interactions between the most-sensitive dorsal olfactory receptors and G-proteins. Receptor Clin. Invest. 3, e1117. 10.14800/rci.1117

[B33] Sato T. Kobayakawa R. Kobayakawa K. Emura M. Itohara S. Kizumi M.. (2015). Supersensitive detection and discrimination of enantiomers by dorsal olfactory receptors: evidence for hierarchical odour coding. Sci. Rep. 5, 14073. 10.1038/srep14073 26361056 PMC4566093

[B34] Sato T. Matsukawa M. Furudono Y. (2014). Algorithm of odor information processing. Oyo-Butsuri 83, 43–47. 10.11470/oubutsu.83.1_43

[B35] Sato T. Matsukawa M. Mizutani Y. Iijima T. Matsumura H. (2018). Initial, transient, and specific interaction between G protein-coupled receptor and target G protein in parallel signal processing: a case of olfactory discrimination of cancer-induced odors. Med. Res. Arch. 6, 1801. 10.18103/mra.v6i9.1801

[B36] Sato T. Nonomura M. Yoneda K. Mizutani S. Mizutani Y. (2021). Prostate cancer-induced changes in urinary odors at biomarker concentrations of ppq with validation by sniffer mouse behavioural assays. Int. J. Cancer Sci. Therapy 3, 2–17. 10.31487/j.IJCST.2021.01.02

[B37] Schaefer M. L. Yamazaki K. Osada K. Restrepo D. Beauchamp G. K. (2002). Olfactory fingerprints for major histocompatibility complex-determined body odors II: relationship among odor maps, genetics, odor composition, and behavior. J. Neurosci. 22, 9513–9521. 10.1523/JNEUROSCI.22-21-09513.2002 12417675 PMC6758037

[B38] Serizawa S. Miyamichi K. Sakano H. (2004). One neuron–one receptor rule in the mouse olfactory system. Trends Genet. 20, 648–653. 10.1016/j.tig.2004.09.006 15522461

[B39] Sharpe L. T. Stockman A. Jägle H. Nathans J. (1999). Opsin genes, cone photopigments, color vision, and color blindness, in Color Vision from Genes to Perception, eds Gegenfurtner K. R. Sharpe L. T. (Cambridge, UK: Cambridge University Press), 3–52.

[B40] Takahashi Y. K. Nagayama S. Mori K. (2004). Detection and masking of spoiled food smells by odor maps in the olfactory bulb. J. Neurosci. 24, 8690–8694. 10.1523/JNEUROSCI.2510-04.2004 15470134 PMC6729973

[B41] Varnet-Maury E. Polak E. H. Demael A. (1984). Structure–activity relationship of stress-inducing odorants in the rat. J. Chem. Ecol. 10, 1007–1018. 10.1007/BF00987509 24318845

[B42] Wiechert M. T. Judkewitz B. Rieche H. Friedrich R. W. (2010). Mechanisms of pattern decorrelation by recurrent neuronal circuits. Nat. Neurosci. 13, 1003–1010. 10.1038/nn.2591 20581841

[B43] Yamazaki K. Boyse E. A. Bard J. Curran M. Kim D. Ross S. R.. (2002). Presence of mouse mammary tumor virus specifically alters the body odor of mice. Proc. Natl. Acad. Sci. U.S.A. 99, 5612–5615. 10.1073/pnas.082093099 11929982 PMC122818

