# Supplementary material for: Hierarchical Elemental Odor Coding for Fine Discrimination Between Enantiomer Odors or Cancer-Characteristic Odors
Source: Front Behav Neurosci. 2022 Apr 22;16:849864. doi: 10.3389/fnbeh.2022.849864 (PMC9074825; doi:10.3389/fnbeh.2022.849864)
Supplement: Supplementary file 1 [file Data_Sheet_1.pdf]

# *Supplementary Material*

## 1 Supplementary Data

## 2 Hierarchical elemental information coding

### 2.1 Hierarchical elemental color coding

Continued from the main text.

In this system, the sign of signals determines one of two opponent colors for each of Y/B and R/G channels. We have realized this principle as we cannot see yellowish blue or reddish green at a single spot.

A more detailed description would be helpful to understanding the principle of elemental color coding. In Supplementary Fig. S1B, the bipolar cells (yellow) summate signals from S, M, and L cone cells, but cannot discriminate isointense total signals which would vary in dominance of S, M, and L signals. A subtraction between receptor signals with overlapped tuning specificities could be essential to reduce redundant information and extract receptor-unique information such as R and G elemental colors for L and M cone cells, respectively. In contrast, an addition of receptor signals with similar tuning specificities could be essential to extract receptor-common information such as Y elemental color for L and M cone cells, by relatively increased signals around the peaks of the tuning specificities. Thus, the retinal ganglion cells with signal subtraction between different receptors could be the first neurons that discriminate elemental colors in the visual pathway. Most retinal ganglion cells transfer their output signals to neurons in the lateral geniculate nucleus (LGN), where is the relay point of visual information from the retina to the primary visual cortex. In the LGN, +L [R], +M [G], L+M [Y], +S [B], +S−(L+M) [+B−Y], −S+(L+M) [+Y−B], +L−M [+R−G], +M−L [+G−R] cells [color tuning] and so on are observed (De Valois et al. 1958; Wiesel and Hubel 1966; Dacey and Lee 1994; Reid and Shapley 2002; De Valois 2004; Field et al. 2007; Conway 2009).

### 2.2 Hierarchical elemental odor coding

Continued from the main text.

Next, we asked whether or not all of the most sensitive ORs could activate the feedforward inhibition for extracting elemental odors. This difficult question could be resolved into elemental factors which sequentially appear in the molecular and cellular steps of odor information processing. Although more various types of ORs are activated as the concentration of a given odorant increases on the olfactory epithelium from the onset to the peak of the nasal inspiratory flow, we recognize a consistent odor for the odorant after we perceive the odor. This fact indicates that the signals of initially activated ORs could govern to represent an odor for a given odorant. To address the above question, we examined four features, that is, (1) rapid and specific interaction between subtypes of ORs and $G_{\alpha olf}$, (2) differences in receptor codes for enantiomeric odorants with similar but different elemental odors, (3) differences in temporal orders of input signals of activated ORs to the brain, and (4) effects of OR deletion on odor discrimination.

Regarding the first issue, a point mutation analysis of helix 8 of an OR S6 revealed that Glu at the 2nd residue of helix 8 is essential for rapid cellular response via specific interaction with a chimeric $G_{\alpha 15\text{-}olf}$ (chimeric $G_{\alpha 15}$ with the C-terminal six amino acids of $G_{\alpha olf}$ instead of its own C-terminal six amino acids, Hamana et al. 2010; Kawasaki et al. 2015). Helix 8 in the C-terminal region of GPCR is an amphipathic alpha helix. The hydrophobic side of helix 8 forms a hydrophobic core with TM1–2 on the cytosolic surface of membrane (Kawasaki et al. 2015). The acidic 2nd residue of Glu on the hydrophilic side of helix 8 is stabilized at the N-terminal corner of helix 8 and the membrane surface by the hydrophobic core and is faced with a widened cavity between TM3 and outwardly moved TM6, through which the $G_{\alpha}$ C-terminal α5 would get access to cytosolic inside of GPCR (Sato et al. 2016b, 2018; Sato 2019).

In fact, onset latencies of $Ca^{2+}$ responses in HEK293 cells co-expressing helix-8-2nd-Glu OR-S6 and nonspecific $G_{\alpha 15}$ (25.0 ± 9.2 s (mean ± S.D.) for 100 μM nonanedioic acid (cc9)) varied ca. 2-fold more widely than those of helix-8-2nd-Glu OR-S6 and specific $G_{\alpha 15\text{-}olf}$ (12.5 ± 7.9 s for 100 μM cc9) (Hamana et al. 2010). This large variability of onset latencies in cells co-expressing helix-8-2nd-Glu OR-S6 and nonspecific $G_{\alpha 15}$ resulted in ca. 2-fold decreased peak height in averaged cellular response curves with multiple peaks. $Ca^{2+}$ responses in HEK293 cells co-expressing helix-8-2nd-Glu OR-S83 and nonspecific $G_{\alpha 15}$ also demonstrated a large variability in peak heights and peak latencies of averaged response curves, whereas those of helix-8-2nd-Glu OR-S83 and specific $G_{\alpha 15\text{-}olf}$ demonstrated rapidly growing single-peaked $Ca^{2+}$ responses (Hamana et al. 2010). HEK293 cells co-expression with specific $G_{\alpha 15\text{-}olf}$, helix-8-2nd-Asp ORs also displayed a large variability in onset latencies in $Ca^{2+}$ responses (our unpublished data). These large variability in onset latencies of helix-8-2nd-Asp ORs would induce trial-by-trial variability (that is, sniffing-by-sniffing variability) in temporal order of input signals of cognate and/or non-cognate ORs to the OB, likely leading to different odor information processing for the identical odor stimulus. This means that onset-latency-variable signals of helix-8-2nd-Asp ORs would cause sniffing-by-sniffing variability in perceived odors if helix-8-2nd-Asp ORs are key ORs for odor representation in the olfactory cortex. In contrast, more synchronized inputs of helix-8-2nd-Glu ORs likely lead to consistent odor information processing for hierarchical elemental odor coding with homeostatic odor perception for a wide range of odor concentrations and odor qualities.

Among non-olfactory GPCRs for identical best agonists, 87% conserved the 2nd residues of helix 8 for each of G protein types (Sato 2019). For example, Gln for opsins/rhodopsin, Asp for β-adrenergic receptors binding to $G_{\alpha s}$, Glu for dopamine receptor 2, 3, 4 binding to $G_{\alpha i}$. In contrast, ORs are classified into three groups with Glu, Gln, or Asp at the 2nd position of helix 8 (Sato et al. 2016b, 2018; Sato 2019). In addition to the above results, the limited data support that the helix-8-2nd-Glu ORs are likely key ORs as determinants of hierarchical elemental odor coding via feedforward inhibition (Sato et al. 2016c). Further study is required to elucidate respective roles of helix-8-2nd-Glu, helix-8-2nd-Gln, and helix-8-2nd-Asp ORs in odor sensitivity and representation. Next, we go on to differences in receptor codes for enantiomeric *R*(−)- and *S*(+)-carvones with similar but different elemental odors.

Receptor codes for *R*(−)- and *S*(+)-carvones have been reported by a random sampling of 2.5 times of all ORs by using calcium imaging of isolated murine OSNs (Hamana et al. 2003). These receptor codes are not complete (a quarter to a half of all carvone ORs), but well describe a key principle and differences in characteristics of ORs between *R*(−)- and *S*(+)-carvones. These ORs are classified into three tuning specificities of *R*(−)-carvone-sensitive ones, *S*(+)-carvone-sensitive ones, and *R*(−)-

/*S*(+)-carvone-equi-sensitive ones, which would contribute to signals of *R*(−)-carvone-unique, *S*(+)-carvone-unique, and *R*(−)-/*S*(+)-carvone-common elemental odors, respectively (Supplementary Figure S2C, D) (Sato et al. 2007, 2015, 2016c, 2018). Among the 15 carvone ORs, eight *R*(−)-/*S*(+)-carvone-equi-sensitive ORs are identically activated in the order of sensitivity between *R*(−)- and *S*(+)-carvones, whereas *R*(−)- and *S*(+)-carvone-sensitive ORs are differently activated in the order of relative sensitivity between *R*(−)- and *S*(+)-carvones (Supplementary Figure S2C (*R*(−)-carvone) and S2D (*S*(+)-carvone)). The input signal intensity of each OR to the OB glomerulus would be proportional to the number of OSNs expressing the respective ORs. This means that a total signal intensity is greater for *R*(−)-/*S*(+)-carvone-equi-sensitive ORs than for *R*(−)- or *S*(+)-carvone-sensitive ORs (eight vs. five or two ORs with an identical factor of 2–4-times for three tuning types of ORs). Notably, the car-*5** OR was expressed in the OSN most sensitive to *R*(−)-carvone enantiomer (Hanama et al. 2003), but the OSNs expressing the car-*5** OR was relatively small in number (Sato et al. 2015). In addition, 11 of the 15 carvone ORs are expressed in the dorsal zone of the olfactory epithelium (marked by the cross) and mostly helix-8-2$^{nd}$-Glu ORs (marked by E), including the car-*5** (Sato et al. 2015). These facts suggest that the most sensitive and dorsal helix-8-2$^{nd}$-Glu ORs could first activate the feedforward inhibitory signals via the sensitive pathway for enhancing *R*(−)-carvone-unique elemental odors against *R*(−)-/*S*(+)-carvone-common elemental odors in the aPC pyramidal cells (Figure 1B) (Sato et al. 2016c, 2018, 2019). Next, we discuss how the olfactory system extracts elemental odors for carvone enantiomers or odor mixtures and then describe how a deletion of all dorsal ORs changes carvone odor discrimination.

We would like to discuss the need for addition and subtraction between signals from multiple ORs. It has been shown in a number of studies that odor compounds can be discriminated already on the level of OB neurons (for example, Cury and Uchida 2010; Shusterman et al 2011). However, even on the level of ORs, odor compounds can be discriminated in given-compound-specific ORs but not in non-specific ORs, leading to compound-discriminating OB neurons and compound-non-discriminating OB neurons via the convergent glomerular input from single-type ORs. In OB neurons receiving signals from sensitive helix-8-2$^{nd}$-Glu ORs, temporal patterns of the responses likely look tightly time-locked to the sniff phase, as observed previously (Cury and Uchida 2010; Shusterman et al 2011). However, even after lateral inhibition slightly sharpen tuning specificities in the OB, redundancy of odor tuning specificities of the OB mitral cells would be too high to represent differences in relative intensities of fresh, sweet, and herbous elemental odors between *R*(−)- and *S*(+)-carvones. To extract elemental odors by reducing information redundancy between odor tuning specificities of OR signals, the addition and subtraction between signals from multiple cognate and non-cognate ORs would be required in the third neurons in the olfactory pathway. At present, it is unknown whether elemental odors are R/G–Y/B-type or R–G–Y–B-type, that is, highly opponent or moderately opponent between some of unique or common elemental odors.

We also discuss how multiple compounds in a mixture of two odors generate a profile of elemental odors as a perceived odor. As described in the main text, a mixture of rose and TMT odors reduced the plasma ACTH which was induced by TMT odor in fear stress responses (Matsukawa et al. 2011). In contrast, a mixture of *S*(+)-carvone and TMT odors did not reduce the plasma ACTH (Murakami et al. 2012). We interpreted these contrasted results as non-TMT odor and TMT-related odor, respectively, in the odor mixtures, where the rose odor and TMT odor are alternatively dominant in a hierarchy of elemental odors. The most sensitive key (likely helix-8-2$^{nd}$-Glu) ORs to compounds of mixture would govern the dominancy of elemental odors in a perceived odor. It is possible but less likely that the perceived odor of the rose and TMT mixture would be completely distinct from both rose and TMT odors.

Moreover, relative and fingerprint-like increases in eight biomarkers added the olfactory cue of prostate cancer to the post-PR urine sample (See the section 4 in the main text). At present, it is unknown whether the olfactory cue of prostate cancer by eight biomarkers is comprised of a single elemental odor or multiple elemental odors in certain relative intensities. Considering the semi-logarithmic increase in perceptual ambiguity of the learned prostate cancer cue around the discrimination threshold, key ORs for the dominant elemental odor in the prostate cancer cue would be one or a few and the decrease in the biomarker concentrations would semi-logarithmically weaken the intensity of the dominant elemental odor. Thus, the hierarchical elemental odor coding scheme provides a consistent explanation for nuanced and various experimental results.

## 3 Genetic ablation of all dorsal olfactory receptors impairs enantiomer odor discrimination and sensitivities to some odorants

Continued from the main text.

The genetic ablation of all dorsal ORs causes more marked reduction of $10^{14}$-fold in odor discrimination between (−)- vs. (+)-wine lactone enantiomers with (+)-enantiomer-specific reduction of $10^{8}$-fold in detection sensitivity (Supplementary Figure S2A) (Sato et al. 2015). Notably, ΔD mice show a $10^{8}$-fold higher detection sensitivity to (−)-wine lactone than (+)-wine lactone. Similarly to ΔD mice, humans also show a $10^{8}$-fold higher detection sensitivity to (−)-wine lactone than (+)-wine lactone (Supplementary Figure S2B) (Guth 1996; Kraft & Mannschrech 2010). This identical difference in detection sensitivities for wine lactone enantiomers between ΔD mice and humans suggests that humans had also lost the most sensitive dorsal ORs for (+)-wine lactone during evolution as ΔD mice.

In another example, key ORs of human OR5AN1 and murine OR215-1 for musk odor (Sato-Akuhara et al. 2016) are also helix-8-2$^{nd}$-Glu ORs and the murine OR215-1 is dorsal OR (Sato et al. 2018). The deletion of OR215-1 resulted in $10^{2}$–$10^{3}$-fold reduction in sensitivity to musk odor (Sato-Akuhara et al. 2016). In contrast, photo-activation of the most sensitive helix-8-2$^{nd}$-Asp OR for TMT, Olfr1019, did not induce a marked increase in plasma ACTH and the deletion of Olfr1019 could not completely impair 10%-TMT-induced immobility of targeted mice (Saito et al. 2017). The most sensitive helix-8-2$^{nd}$-Glu OR for TMT, Olfr30, is less sensitive than the most sensitive helix-8-2$^{nd}$-Asp OR, Olfr1019, suggesting a compensatory role of helix-8-2$^{nd}$-Asp ORs in odor perceptual sensitivity.

In addition, in the fourth OR group of trace amine-associated receptors (TAARs) with helix-8-2$^{nd}$-Trp, the deletion of the most sensitive dorsal TAAR4 for phenylethylamine elevated the EC50 by ca. 10 times (Dewan et al. 2018). In rats, a behavioral assay emphasized the importance of early OR signals for rapid odor discrimination in a single sniff (<200 ms) (Uchida & Mainen, 2003). Moreover, a greater importance of earlier-activated OR signals has been reported in synthetic optogenetic odor-evoked responses in mice (Chong et al. 2020). These facts provide evidence for our hierarchical elemental odor coding scheme where sensitive (and dorsal) helix-8-2$^{nd}$-Glu ORs are key ORs for stimulus-driven characteristic elemental odor enhancing system.

## 4 Bladder- and prostate-cancer odor detection and discrimination

See the main text.

## 5 Shared features between humans and mice

First, the helix-8 $2^{nd}$ residues of ORs are 91% (243/268) identical between humans and mice in class-I (93% (39/42), Glu and Gln) and class-II (90% (204/226), Glu, Gln and Asp) ORs, and TAARs (100% (5/5), Trp) (Sato et al. 2018). In addition, occupancies of helix-8-$2^{nd}$ Glu in class I and II ORs (23% (24%) and 47% (42%), respectively, in humans (mice)), Gln (69% (67%) and 7% (8%), respectively, in humans (mice)), and Asp (0% (0%) and 42% (48%), respectively, in humans (mice)) are almost identical between humans and mice (Supplementary Table ST1) (Sato 2019). Considering high sequence variability within and between family members of GPCRs, these conserved helix-8-$2^{nd}$ residues could confer distinct functional roles of distinct subgroups of ORs in odor information processing.

If the color coding and odor coding would share a common principle in neural information processing with GPCR-detecting signals, humans and mice could share common features in elemental odor coding. Furudono et al. found three similar groups of nine odorants among 12 odorants between human-perceived odors and murine receptor codes and a different grouping of remaining three odorants (Supplementary Figure S3) (Furudono et al. 2009; Sato et al. 2014; Sato 2019). Interestingly, three sets of odorants for vanilla (three odorants), creamy (two odorants), and cinnamon odors (two odorants), respectively, in human perception activated several cognate murine ORs with one of corresponding three tuning specificities where each OR is activated only by corresponding three, two, and two odorants, respectively (Supplementary Figure S3A, B) (Furudono et al. 2009; Sato et al. 2014; Sato 2019). Based on the ratio (1.3) of sampling number to the repertoire of ca. 1,130 receptors, signals from an estimated four (profile 11), five (profile 32), and five (profile 39) different and cognate ORs are likely integrated and represent vanilla, creamy and cinnamon elemental odors, respectively, in the aPC pyramidal cells via feedforward inhibition activated by earlier signals from a helix-8-$2^{nd}$-Glu one of the cognate ORs (Sato et al. 2014; Sato 2019). In addition, humans perceive similar odors of balsamic cinnamon or rooty wintergreen between three odorants of t-cinnamic aldehyde, cinnamic alcohol, and methyl salicylate, which are closely arranged (Supplementary Figure S3C). In contrast, these three odorants are much separately arranged in murine receptor codes (Supplementary Figure S3B). This difference is attributable to a larger repertoire of murine ORs than human ORs, meaning that more different tuning ORs responsive to one of the three odorants enable mice to discriminate them each other.

## 6 Future studies

In class A GPCRs to which ORs belong, a stable interaction between a GPCR and its specific G protein has been revealed (Rasmussen et al. 2011) and leaded to a discovery of shared features of changes in intramolecular interactions between inactive and active states of GPCRs (Huang et al. 2015). The shared set of interacting residue pairs in the stable interactions between GPCRs and G proteins are likely applicable to a stable interaction between an OR−S6 and $G_{\alpha olf}$ (Sato 2019). Compared to entirely low sequence homology of GPCRs, the highly conserved helix-8 $2^{nd}$ residue in groups for identical best ligands and specific G protein types would confer distinct functional roles of helix-8-$2^{nd}$-resudue classified GPCRs via the initial and specific interaction between GPCR and G protein in their signaling pathways. Based on differences in rapid and specific interaction with $G_{\alpha olf}$, functional roles of helix-8-$2^{nd}$-Glu, Gln, Asp, and Trp in a hierarchical elemental odor coding scheme will be elucidated in future. There are several ORs with helix-8-$2^{nd}$ Lys/His in both of class I and II ORs (Sato 2019). It is possible that the positively charged helix-8-$2^{nd}$-Lys/His are essential to specific interaction with inhibitory $G_{\alpha i}$ in cAMP second messenger system for a distinct functional

role of these minor ORs such as off responses. Based on these principles, we will understand the details of the complicated odor information processing for behavioral controls in the olfactory system with decorrelation of strongly overlapped OR signals in postsynaptic circuitry in the OB (Fredrich et al. 2009; Niessing et al. 2010; Wiechert et al. 2010; Gilra et al. 2015) and the sensitive and less-sensitive OR signal routes from the OB to the aPC (Matsutani et al. 1989; Igarashi et al. 2012).

## 7 References

Calkins DJ. (2004). Linking retinal circuits to color opponency. In Chalupa LM & Werner JS (eds), *The visual neurosciences*. The MIT Press, Cambridge, MA, U.S.A. pp. 989–1002.

Chong E, Moroni M, Wilson C, Shoham S, Panzeri S, Ringerg D. (2020). Manipulating synthetic optogenetic odors reveals the coding logic of olfactory perception. *Science* 368, eaba2357. doi: 10.1126/science.aba2357.

Conway BR. (2009). Color vision, cones, and color-coding in the cortex. *The Neuroscientist* 15, 274–290. doi: 10.1177/1073858408331369.

Cury KM, Uchida N. (2010). Robust odor coding via inhalation-coupled transient activity in the mammalian olfactory bulb. *Neuron* 68, 570–585. doi: 10.1016/j.neuron.2010.09.040.

Dacey DM, Lee BB. (1994). The 'blue-on' opponent pathway in primate retina originates from a distinct bistratified ganglion cell type. *Nature* 367, 713–715. doi: 10.1038/367731a0.

De Valois RL, Smith CJ, Kitai ST, Karoly AJ. (1958). Response of single cells in monkey lateral geniculate nucleus to monochromatic light. *Science* 127, 238–239.

De Valois RL. (2004). Neural coding of color. In Chalupa LM & Werner JS (eds), *The visual neurosciences*. The MIT Press, Cambridge, MA, U.S.A. pp. 1003–1016.

Dewan A, Cichy A, Zhang J, Miguel K, Feinstein P, Rinberg D, et al. (2018). Single olfactory receptors set odor detection thresholds. *Nat Commun.* 9, 2887. doi: 10.1038/s41467-018-05129-0.

Field GD, Sher A, Gauthier JL, Greschner M, Shlens J, Litke AM, et al. (2007). Spatial properties and functional organization of small bistratified ganglion cells in primate retina. *J Neurosci* 27, 13261–13272. doi: 10.1523/JNEUROSCI.3437-07.2007.

Friedrich RW, Yaksi E, Judkewitz B, Wiechert MT. (2009). Processing of odor representations by neuronal circuits in the olfactory bulb. *Ann NY Acad Sci.* 1170, 293–297. doi: 10.1111/j.1749-6632.2009.04010.x.

Furudono Y, Sone Y, Takizawa K, Hirono J, Sato T. (2009). Relationship between peripheral receptor code and perceived odor quality. *Chem Senses* 31, 151–159. doi: 10.1093/chemse/bjn071.

Gilra A, Bhalla US. (2015). Bulbar microcircuit model predicts connectivity and roles of interneurons of odor coding. *PLoS ONE* 10, e0098045. doi: 10.1371/journal.pone.0098045.

Guth H. (1996). Determination of the configuration of wine lactone. *Helv Chem Acta.* 79, 1559–1571. doi: 10.1002/hlca.19960790606.

Hamana H, Hirono J, Kizumi M, Sato T. (2003). Sensitivity-dependent hierarchical receptor codes for odors. *Chem Senses* 28, 87–104. doi: 10.1093/chemse/28.2.87.

Hamana H, Li S, Breuils L, Hirono J, Kizumi M, Sato T. (2010). Heterologous functional expression system for odorant receptor. *J Neurosci Meth.* 185, 213–220. doi: 10.1016/j.jneumeth.2009.09.024.

Huang W, Manglik A, Venkatakrishnan AJ, Laeremans T, Feinberg EN, Sanborn AL, et al. (2015). Structural insights into μ opioid receptor activation. *Nature* 524, 315−321. doi: 10.1038/nature14886.

Igarashi KM, Ieki N, An M, Yamaguchi Y, Nagayama S, Kobayakawa K, et al. (2012). Parallel mitral and tufted cell pathways route distinct odor information to different targets in the olfactory cortex. *J Neurosci.* 32, 7870–7885. doi: 10.1523/JNEUROSCI.0154-12.2012.

Kawasaki T, Saka T, Mine S, Mizohata E, Inoue T, Matsumura H, et al. (2015). The N-terminal acidic residue of the cytosolic helix 8 of an odorant receptor is responsible for different response dynamics via G-protein. *FEBS Lett.* 589, 1136−1142. doi: 10.1016/j.febslet.2015.03.025.

Kraft P, Mannschreck A. (2010). The enantioselectivity of odor sensation: some examples for undergraduate chemical course. *J Chem Educ.* 87, 598–603. doi: 10.1021/ed100128v.

Matsutani S, Senba E, Tohyama M. (1989). Terminal field of cholecystokinin-8-like immunoreactive projection neurons of the rat main olfactory bulb. *J Comp Neurol.* 285, 73–82. doi: 10.1002/cne.902850107.

Murakami T, Matsukawa M, Katsuyama N, Imada M, Aizawa S, Sato T. (2012). Stress-related activities induced by predator odor may become indistinguishable by hinokitiol odor. *Neuroreport* 23, 1071−1076. doi: 10.1097/WNR.0b013e32835b373b.

Niessing J, Friedrich RW. (2010). Olfactory pattern classification by discrete neuronal network states. *Nature* 465, 47–52. doi: 10.1038/nature08961.

Rasmussen SG, DeVree BT, Zou Y, Kruse AC, Chung KY, Kobilka TS, et al. (2011). Crystal structure of the β2 adrenergic receptor-Gs protein complex. *Nature* 477, 549−555. doi: 10.1038/nature10361.

Reid RC, Shapley RM. (2002). Space and time maps of cone photoreceptor signals in macaque lateral geniculate nucleus. *J Neurosci* 22, 6158–6175. doi: 10.1523/JNEUROSCI.22-14-06158.2002.

Saito H, Nishizumi H, Suzuki S, Matsumoto H, Ieki N, Abe T, et al. (2017). Immobility responses are induced by photoactivation of single glomerular species responsive to fox odour TMT. *Nat Commun.* 8, 16011. doi: 10.1038/ncomms16011.

Sato T, Ishikawa T, Shimizu A, Hirono J, Hamana H, Iijima T. (2007). Molecular basis of odor discrimination in olfactory system. *Seitai-no-Kagaku* 58, 264–268 (Japanese).

Sato T, Matsukawa M, Furudono Y. (2014). Algorithm of odor information processing. *Oyo-Butsuri* 83, 43–47 (Japanese).

Sato T, Kobayakawa R, Kobayakawa K, Emura M, Itohara S, Kizumi M, et al. (2015). Supersensitive detection and discrimination of enantiomers by dorsal olfactory receptors: evidence for hierarchical odour coding. *Sci Rep.* 5, 14073. doi: 10.1038/srep14073.

Sato T, Kawasaki T, Mine S, Matsumura H. (2016b). Functional role of the C-terminal amphipathic helix 8 of olfactory receptors and other G protein-coupled receptors. *Int J Mol Sci.* 17, E1930. doi: 10.3390/ijms17111930.

Sato T, Kobayakawa R, Kobayakawa K, Emura M, Itohara S, Kawasaki T, et al. (2016c). Supersensitive odor discrimination is controlled in part by initial transient interactions between the most-sensitive dorsal olfactory receptors and G-proteins. *Receptor Clin Invest* 3, e1117. doi: 10.14800/rci.1117.

Sato T, Matsukawa M, Mizutani Y, Iijima T, Matsumura H. (2018). Initial, transient, and specific interaction between G protein-coupled receptor and target G protein in parallel signal processing: a case of olfactory discrimination of cancer-induced odors. *Med Res Arch* 6, 1801. doi: 10.18103/mra.v6i9.1801.

Sato T. (2019). Conserved 2nd residue of helix 8 of GPCR may confer the subclass-characteristic and distinct roles through a rapid initial interaction with specific G proteins. *Int J Mol Sci* 20, 1752. doi: 10.3390/ijms20071752.

Sato-Akuhara N, Horio N, Kato-Namba A, Yoshikawa K, Niimura Y, Ihara S, et al. (2016). Ligand specificity and evolution of mammalian musk odor receptors: effect of single receptor deletion on odor detection. *J Neurosci.* 36, 4482–4491. Doi: 10.1523/JNEUROSCI.3259-15.2016.

Sharpe LT, Stockman A, Jägle H, Nathans J. (1999). Opsin genes, cone photopigments, color vision, and color blindness. In Gegenfurtner KR & Sharpe LT (eds), *Color vision from genes to perception*. Cambridge University Press, Cambridge, UK. pp. 3–52.

Shusterman R, Smear MC, Koulakov AA, Rinberg D. (2011). Precise olfactory responses tile the sniff cycle. *Nat Neurosci.* 14, 1039–1044. doi: 10.1038/nn.2877.

Uchida N, Mainen ZF. (2003). Speed and accuracy of olfactory discrimination in the rat. *Nat Neurosci.* 6, 1224–1229. doi: 10.1038/nn.1142.

Wiechert MT, Judkewitz B, Rieche H, Friedrich RW. (2010). Mechanisms of pattern decorrelation by recurrent neuronal circuits. *Nat Neurosci.* 13, 1003–1010. doi: 10.1038/nn.2591.

Wiesel TN, Hubel DH. (1966). Spatial and chromatic interactions in the lateral geniculate body of the rhesus monkey. *J Neurophysiol* 29, 1115–1156. doi: 10.1152/jn.1966.29.6.1115.

# 8 Supplementary Figures and Tables

## 8.1 Supplementary Table

**Supplementary Table ST1**. Conserved helix-8 2nd residues of class-I, class-II ORs and TAARs between humans and mice (Sato 2019).

| | Helix-8 second residue | | | | | | | | | | |
|---|---|---|---|---|---|---|---|---|---|---|---|
| | all | Glu | Gln | Asp | Asn | Trp | His | Lys | Arg | misc | Identity |
| Human class-I ORs ($G_{olf}$) | 52 | 12 | 36 | 0 | 0 | 0 | 1 | 1 | 0 | 2 | |
| | 100% | 23% | 69% | 0% | 0% | 0% | 2% | 2% | 0% | 4% | 93% 39/42 |
| Murine class-I ORs ($G_{olf}$) | 123 | 29 | 83 | 0 | 0 | 0 | 0 | 5 | 0 | 6 | |
| | 100% | 24% | 67% | 0% | 0% | 0% | 0% | 4% | 0% | 5% | |
| Human class-II ORs ($G_{olf}$) | 333 | 156 | 22 | 139 | 1 | 0 | 2 | 6 | 0 | 7 | |
| | 100% | 47% | 7% | 42% | 0% | 0% | 1% | 2% | 0% | 2% | 90% 204/226 |
| Murine class-II ORs ($G_{olf}$) | 979 | 409 | 75 | 467 | 7 | 0 | 1 | 6 | 0 | 14 | |
| | 100% | 42% | 8% | 48% | 1% | 0% | 0% | 1% | 0% | 1% | |
| Human TAAR ORs ($G_s$, $G_q$, $G_i$) | 6 | 0 | 0 | 0 | 0 | 6 | 0 | 0 | 0 | 0 | |
| | 100% | 0% | 0% | 0% | 0% | 100% | 0% | 0% | 0% | 0% | 100% 5/5 |
| Murine TAAR ORs ($G_s$, $G_q$, $G_i$) | 15 | 0 | 0 | 0 | 0 | 15 | 0 | 0 | 0 | 0 | |
| | 100% | 0% | 0% | 0% | 0% | 100% | 0% | 0% | 0% | 0% | |
| Total human ORs ($G_{olf}$) | 385 | 168 | 58 | 139 | 1 | 0 | 3 | 7 | 0 | 9 | |
| | 100% | 44% | 15% | 36% | 0% | 0% | 1% | 2% | 0% | 2% | 91% 243/268 |
| Total Murine ORs ($G_{olf}$) | 1102 | 438 | 158 | 467 | 7 | 0 | 1 | 11 | 0 | 20 | |
| | 100% | 40% | 14% | 42% | 1% | 0% | 0% | 1% | 0% | 2% | |

## 8.2 Supplementary Figures

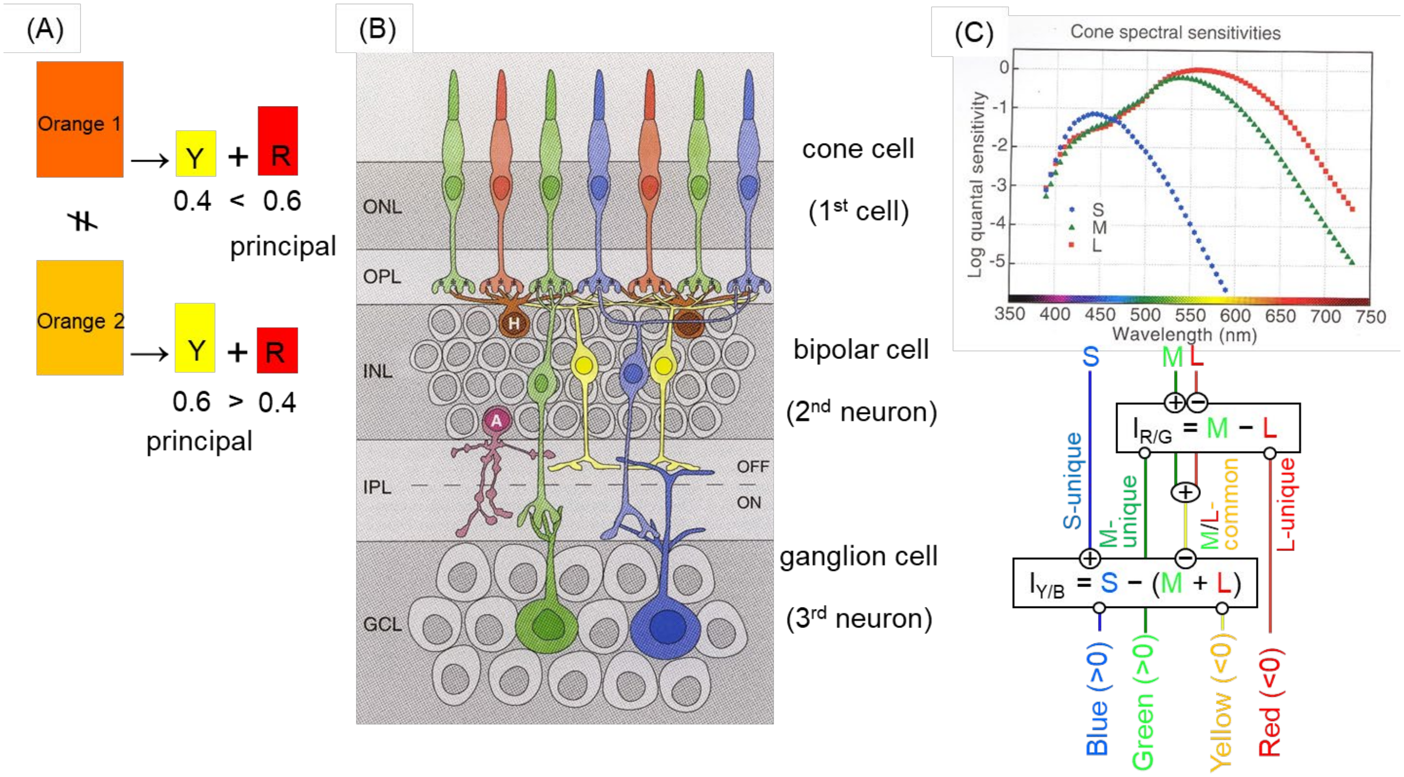


**Supplementary Figure S1.** Hierarchical elemental color coding in color vision. (**A**), Schematic diagram of two distinct orange colors in a hierarchical elemental color coding. The orange colors are composed of yellow and red colors in distinct relative intensities. The elemental colors of yellow and red are essential to discriminate a difference in orange colors. (**B**), Retinal neural pathway from cone cells to ganglion cells (Calkins 2004, Copyright: MIT Press, republished with permission). Ganglion cells are the third cells from cone cells as the first cells that discriminate elemental colors in the visual pathway. The ganglion cell (blue) is the S-ON/(M + L)-OFF ganglion cell for B/Y color opponency receiving signals from S-ON (blue) and (M + L)-OFF bipolar (yellow) cells, whereas the ganglion cell (green) is the midget/P ganglion cell for R/G color opponency which receives excitation from a single midget bipolar cell and inhibition via horizontal (H) and amacrine (A) cells. (**C**), Four elemental colors of R/G and Y/B (I, signal intensity) are extracted by addition (+) and/or subtraction (−) between three receptors of L, M, and S via inhibitory signals (Sharpe et al.1999, Copyright: Cambridge University Press, republished with permission). Blue, green, and red are S-, M-, and L-unique elemental colors, respectively, whereas yellow is the M- and L-common elemental color being represented by addition of signals from L and M.

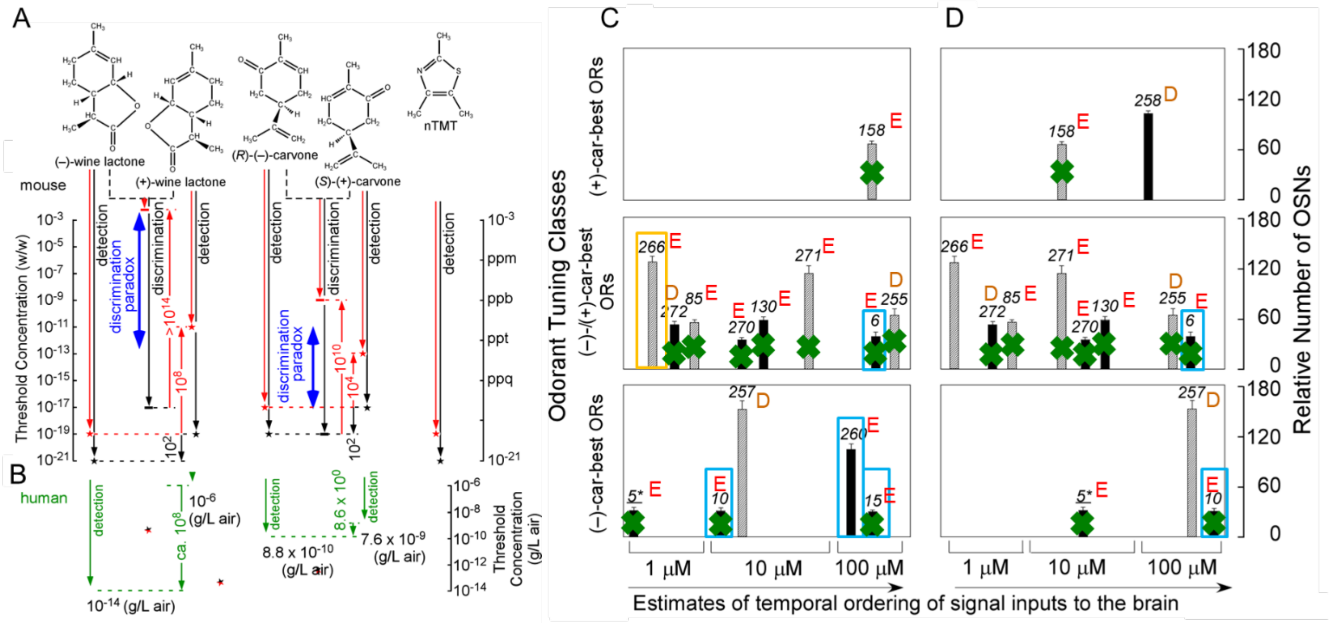


**Supplementary Figure S2.** Genetic ablation of all dorsal olfactory receptors impairs enantiomer odor discrimination (Sato et al. 2015, 2016c, 2018). (**A**), Detection and discrimination thresholds for (−)- and (+)-enantiomers in mice. Wild-type (WT) mice detected and discriminated enantiomers with similar detection (black stars) and discrimination thresholds (black bold bars). In contrast, ΔD mice showed marked and moderate elevation in enantiomer discrimination and (+)-enantiomer detection thresholds, respectively. Although activated ORs differ between carvone enantiomers (**C** vs. **D**), ΔD mice could not discriminate for carvone enantiomers (discrimination paradox). (**B**), Detection thresholds for (−)- and (+)-enantiomers in humans. The difference in detection thresholds between (−)- and (+)-wine lactone was almost the same between ΔD mice and humans. (**C**), Temporal order of *R*(−)-carvone-activated olfactory receptors (ORs). (**D**), Temporal order of *S*(+)-carvone-activated ORs. *Numbers* represent the OR names as car-*5* OR (Olfr1366, the most sensitive and *R*(−)-carvone-sensitive dorsal OR among carvone-activated ORs), car-*266* OR (Olfr1484, the most sensitive *R*(−)/*S*(+)-carvone-common ventral OR, enclosed in the orange rectangle), car-*10* OR (Olfr1234, human ortholog: OR4A15), and so on. ORs marked by the green cross are activated in wild-type (WT) mice, but not in ΔD mice. *the most sensitive OR; E, helix-8-2$^{nd}$-Glu OR; D, helix-8-2$^{nd}$-Asp OR; ORs (enclosed in the blue rectangle) have human orthologous ORs.

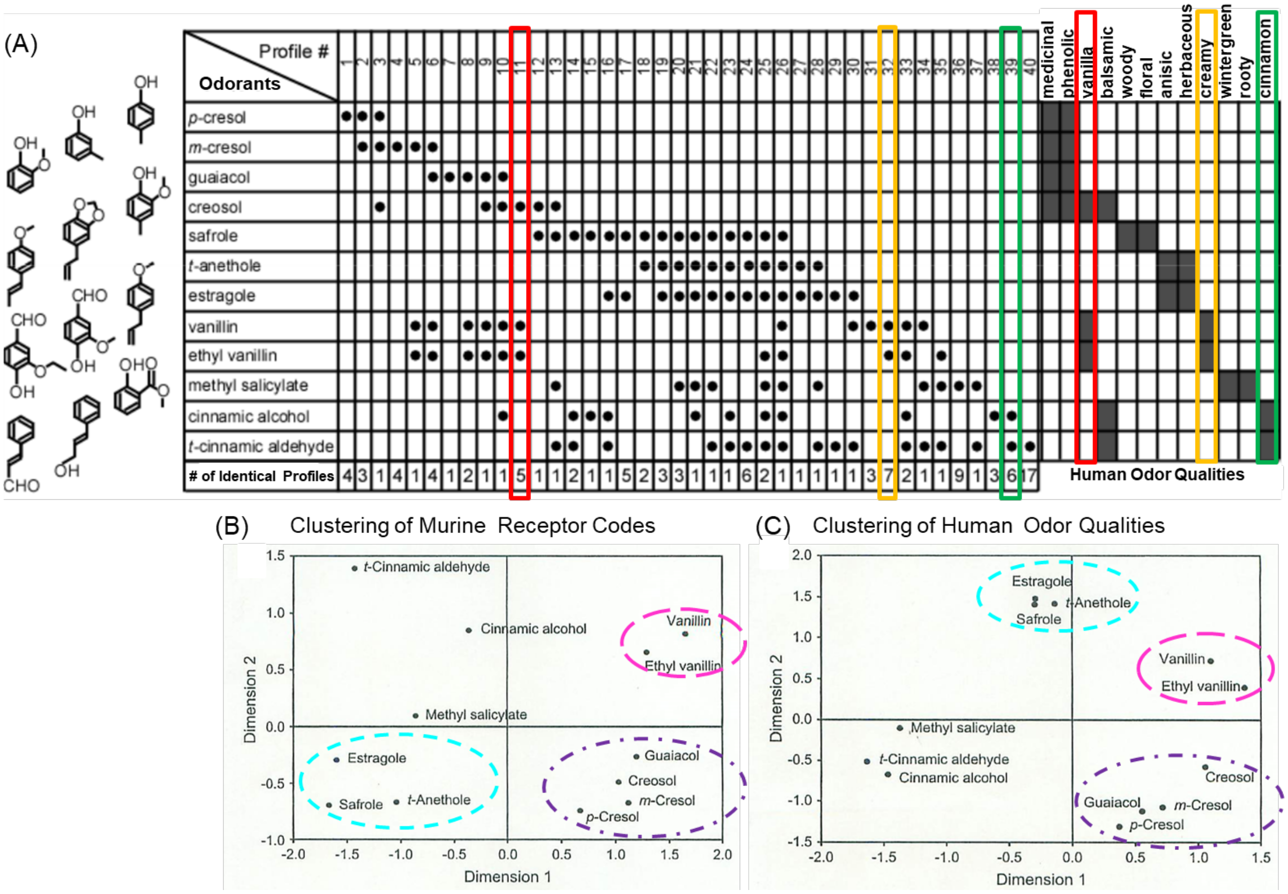


**Supplementary Figure S3.** Murine receptor codes and human perceived odor qualities for 12 odorants (Furudono et al. 2009). (**A**), Murine receptor codes for 12 odorants. Total 1,443 olfactory sensory neurons (OSNs) were tested. 55 OSNs responded to ≥1 of 12 odorants, resulting in 40 different response profiles. Based on the ratio (1.3) of sampling number to the repertoire of ca. 1,130 receptors, five, seven and six murine OSNs for the profile 11, 32 and 39, respectively, which responded only to three, two and two odorants that only evoked vanilla, creamy and cinnamon odors, respectively, in humans were estimated to be comprised of different OSNs expressing one of four, five and five different olfactory receptors, respectively. (**B**), Clustering of murine receptor codes for 12 odorants. Three clusters and three singletons for 12 odorants in murine receptor codes were formed in a multidimensional analysis. (**C**), Clustering of human odor qualities for 12 odorants. Similarly to those of murine receptor codes, human odor qualities for nine of 12 odorants formed three clusters in a multidimensional analysis.
